# The impact of the COVID-19 pandemic on pleural infection incidence: a UK multicentre retrospective analysis

**DOI:** 10.1183/23120541.00206-2022

**Published:** 2022-08-01

**Authors:** Eihab O. Bedawi, Khalil Ur Rehman, Deepan P. Sivakumar, Katie Ferguson, Syed Ajmal, Emma Graham, Rakesh K. Panchal, John P. Corcoran, Kevin G. Blyth, Najib M. Rahman, Alex West

**Affiliations:** 1 Oxford Pleural Unit, Oxford Centre for Respiratory Medicine, Oxford University Hospitals NHS Foundation, Oxford, UK; 2 Oxford NIHR Biomedical Research Centre, University of Oxford, Oxford, UK; 3 Dept of Respiratory Medicine, Guy's and St Thomas’ NHS Foundation Trust, London, UK; 4 Institute of Cancer Sciences, University of Glasgow, Glasgow, UK; 5 Glasgow Pleural Disease Unit, Queen Elizabeth University Hospital, Glasgow, UK; 6 University Hospitals of Leicester NHS Trust, Leicester, UK; 7 University Hospitals Plymouth NHS Trust, Plymouth, UK; 8 Co-first authors; 9 Co-senior authors

## Abstract

The fall in non-coronavirus disease 2019 (non-COVID-19) respiratory viruses, including seasonal influenza, during the pandemic is well reported [1–4]. It is thought to be a result of a combination of social distancing, lockdowns, improved hand hygiene and potentially virus–virus interactions and cross-protection affecting population dynamics. However, as vaccines weaken the transmission of severe acute respiratory syndrome coronavirus 2 (SARS-CoV-2), clinicians remain vigilant for a potential resurgence of other respiratory pathogens and the implications of an ongoing rise in new SARS-CoV-2 variants.

## Abstract 2

**There was a significant reduction in pleural infection incidence, by almost a third, in the year following the start of the #COVID19 pandemic. Public health measures enforced during this period are likely to have played a significant role.** https://bit.ly/3QAPPR9

*To the Editor:*

The fall in non-coronavirus disease 2019 (non-COVID-19) respiratory viruses, including seasonal influenza, during the pandemic is well reported [1–4]. It is thought to be a result of a combination of social distancing, lockdowns, improved hand hygiene and potentially virus–virus interactions and cross-protection affecting population dynamics. However, as vaccines weaken the transmission of severe acute respiratory syndrome coronavirus 2 (SARS-CoV-2), clinicians remain vigilant for a potential resurgence of other respiratory pathogens and the implications of an ongoing rise in new SARS-CoV-2 variants.

A huge rise in pleural infection cases is well documented following the influenza pandemic in 1918 [5] and a recent epidemiological study from Arnold *et al.* [6] also found that for nine of the 10 years studied, the highest annual point incidence of influenza nationally coincided with the highest admission rate for pleural infection.

Pleural effusions have been noted in only a minority of severe COVID-19 cases (up to 5%) [7]. To date, no studies have examined the overall impact of the COVID-19 pandemic on adult pleural infection incidence. This study was therefore planned to assess the impact of the pandemic on incidence and profile of pleural infection.

The study was conducted through a network of geographically diverse specialist pleural units across the UK actively recruiting to a prospective multicentre randomised controlled trial (RCT), the third Multicentre Intrapleural Sepsis Trial (MIST-3; ISRCTN registry identifier ISRCTN18192121). The five centres were continuously screening for cases of pleural infection within their services and had dedicated pleural multidisciplinary team (MDT) meetings that captured and recorded all pleural infections across their services as standard practice.

Two comparative periods were chosen as March 2020 to February 2021 (post-COVID), to represent the study cohort, against the same period before the COVID-19 pandemic (March 2019 to February 2020; pre-COVID) as a control cohort. A retrospective review of screening logs and case notes was conducted.

The primary outcome measure was the difference in incidence (number of confirmed cases) of pleural infection admissions between the two time periods. Secondary outcome measures included the following: comparison of patient demographics; interval between symptoms onset and presentation; incidence of pleural infection during the influenza season; the effect of immunosuppression (patient receiving regular steroids, biological agents or active chemotherapy on admission); radiological evidence of pneumonic consolidation and/or COVID-19 infection (according to the reporting radiologist); and microbiological profile. As clinical outcomes would be skewed by involvement of the study cohort in the MIST-3 study, which involves early randomisation to one of three study intervention arms (standard care, intrapleural fibrinolytics, or an early surgical opinion), these were not evaluated. In the study cohort, data on COVID-19 PCR positivity was also collected. As this was a retrospective analysis, patient consent and research ethics committee approval were not required.

To be included, the patients had to be adults (aged ≥18 years) with a diagnosis of pleural infection based on standard, internationally agreed criteria [8] (identical to those used in large prospective RCTs [9]). These criteria were: 1) a clinical history compatible with pleural infection, and 2) a pleural collection that was either purulent, or Gram stain/culture positive, or acidic with a low pH (<7.2), or had a low pleural fluid glucose (in the absence of an accurate pH measurement), or a septated pleural collection clinically considered most likely secondary to pleural infection.

Statistical analyses were conducted using simple descriptive statistics. Continuous variables were reported as mean and standard deviation. Comparisons of proportions were conducted using the Pearson's Chi-squared test (p<0.05). The analyses were conducted using SPSS (IBM, version 28).

A total of 308 patients were included in the final analysis. In the 1-year pre-COVID period, 184 new cases of pleural infection were identified across the five participating centres *versus* 124 new cases in the 1-year period following the start of the pandemic. This equated to a decrease of 32.61% in admissions between the two years (figure 1).

**FIGURE 1:**
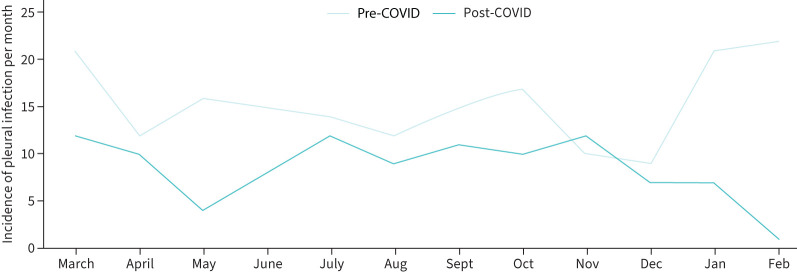
Incidence of pleural infection per month. Pre-COVID: March 2019 to February 2020. Post-COVID: March 2020 to February 2021.

Patient demographics (age and sex distribution, infection setting (hospital-acquired *versus* community-acquired), immunosuppression and median RAPID score (renal, age, purulence, infection source, and dietary factors)) were similar in both groups with no statistically significant differences in the two time periods studied. The median interval between symptoms onset and hospital attendance was slightly longer in the post-COVID cohort (14 *versus* 10 days) but did not reach statistical significance (p=0.16). All patients had computed tomography scans and none of these showed evidence of co-existent COVID-19 pneumonia.

Analysis of pleural infection cases diagnosed during the influenza season (December, January and February) showed 46 out of 184 (25%) in the pre-COVID period, and 15 out of 124 (12.1%) cases during those 3 months in the post-COVID period. This difference was statistically significant (Chi-squared 7.765 (1 df, n=308), p=0.005).

Greater proportions of pleural fluid purulence (49 out of 124 (39.5%) *versus* 50 out of 184 (27.2%); p=0.04) and culture-positive infections (48 out of 124 (38.7%) *versus* 49 out of 184 (26.6%); p=0.03) were observed in the post-COVID period. The species of microorganisms isolated on pleural fluid culture were similar in both cohorts.

SARS-CoV-2 PCR positivity was seen in nine out of 124 (7.25%) patients in the post-COVID cohort.

We present, to our knowledge, the first study examining the impact of COVID-19 on the incidence and profile of pleural infection. Epidemiological studies have suggested that the incidence has steadily increased year-on-year in the last decade [10–12]. In this representative sample of the UK population, covering five geographically diverse areas, our data demonstrate a 32.6% decrease in pleural infection in the year following the start of the COVID-19 pandemic. The higher proportion of purulent and microbiology-positive infections in the post-COVID cohort may have been a result of poorer access to prior antibiotics in the community [13].

The low rate of co-existent COVID-19 PCR positivity (7.25%) and the absence of radiological evidence of COVID-19 pneumonia in any of the pleural infection cases are in keeping with the literature, which suggests that empyema does not appear to be frequently associated with COVID-19 pneumonia [7].

Compared to previous influenza pandemics, it is noteworthy that during the COVID-19 peaks, antibiotics were used sparingly to cover secondary bacterial infections, and this may have helped prevent empyema complications.

The potential role of public health measures in reducing pleural infection incidence is intriguing and one that has not been specifically explored in the existing literature. It is highly likely that decreased social mixing, shielding of older, more vulnerable patients with additional comorbidity, often at increased risk of pleural infection, as well as social distancing measures have had, in combination, a beneficial impact.

There were some limitations to this study. Being retrospective, it is bound by the validity and bias limitations of such a study design. However, the regular prospective screening procedures in place as part of recruitment to the MIST-3 study and the prospective documentation of pleural infection cases through the weekly specialist MDT meetings at the participating centres are likely to have been more robust methods of capturing cases compared to hospital episode statistics or administrative databases. These data only capture patients admitted to hospital and therefore cannot exclude that a proportion of patients chose to be treated in the community to avoid catching COVID-19 in hospital. The impact of vaccinations remains unclear. It also remains to be seen whether pleural infection incidence will return to pre-pandemic levels as enforcement of public health measures is relaxed.

This study demonstrates a reduction in pleural infection incidence, by almost a third, following the start of the COVID-19 pandemic. Potential causes for lower rates of pleural infection may be secondary to reduced community transmission of viruses due to social distancing and use of personal protective equipment in both community and healthcare settings.
